# The impact of early nutritional and immune status assessment on all-cause mortality in patients with intracerebral hemorrhage in the intensive care unit: a retrospective study

**DOI:** 10.3389/fmed.2026.1852107

**Published:** 2026-07-07

**Authors:** Jun Chen, Hui-Zhen Chen, Jun-Feng Lu, Yi-Yi Wu, Jian-Ting Gao

**Affiliations:** 1 Department of Critical Care Medicine, Hangzhou Lin’an Traditional Chinese Medicine Hospital, Hangzhou, China; 2 Department of Critical Care Medicine, Hangzhou Hospital of Traditional Chinese Medicine, Hangzhou, China; 3 Department of Emergency, Hangzhou Hospital of Traditional Chinese Medicine, Hangzhou, China

**Keywords:** HALP score, immune, intracerebral hemorrhage, mortality, nutritional

## Abstract

**Background:**

Recent research has indicated that nutritional and immune status impact neurological outcomes. We assessed the association between the hemoglobin–albumin–lymphocyte platelet (HALP) score and all-cause mortality among patients with intracerebral hemorrhage (ICH).

**Methods:**

We retrospectively collected data from patients with ICH in two cohorts. Kaplan–Meier survival curves and restricted cubic spline (RCS) regression analysis were used to analyze the association between the HALP score and both short- and long-term outcomes. We also developed a risk prediction nomogram and validated it in an external cohort.

**Results:**

An analysis of 925 ICH patients from the MIMIC database revealed 30-day, 90-day, and 365-day mortality rates of 29.08%, 34.49%, and 41.84%, respectively. The Q1 group (HALP < 16.87) exhibited significantly higher short- and long-term mortality than the other groups (all *p* < 0.001). The RCS analysis showed that there was a non-linear correlation between the HALP score and mortality risk. These findings were confirmed in the external validation cohort. The risk model established based on nutritional assessment can effectively identify high-risk ICH patients. The nomogram showed strong sensitivity and specificity in the internal cohort, achieving an area under the receiver operating characteristic (AUROC) curve of 0.825.

**Conclusion:**

Our findings indicate that a lower HALP score is associated with increased mortality in ICH patients. Additionally, we created a straightforward risk stratification tool based on this score to assist clinicians in identifying high-risk individuals.

## 1 Introduction

Intracerebral hemorrhage (ICH) constitutes approximately 15% of all strokes and is responsible for 50% of stroke-related deaths, resulting in approximately 2.8 million fatalities annually worldwide (1, 2). Significant ethnic and socioeconomic disparities affect ICH incidence and outcomes, with Asians experiencing higher rates than other ethnic groups globally (3). The high mortality rate, frequently due to the initial neurological injury and its systemic effects, highlights the critical need for effective prognostic tools.

Several hours after intracerebral hemorrhage (ICH) occurs, a series of neuro-inflammatory responses develops around the hematoma, disrupting immune homeostasis. These reactions not only promote hematoma expansion but also lead to severe catabolism and nutritional depletion, thereby exacerbating clinical outcomes (4, 5). Therefore, in addition to the severity of neurological manifestations, the systemic status of patients with intracerebral hemorrhage—including inflammatory, immune, and nutritional conditions—is gaining increasing attention (6–8).

Increasing focus has been directed toward identifying an optimal laboratory parameter to predict outcomes in ICH patients from both inflammatory and nutritional perspectives. Research has indicated that patients with poor prognosis following acute intracerebral hemorrhage often experience more severe malnutrition than those with better outcomes (7). Hypoalbuminemia, an indicator of malnutrition, has been associated with higher mortality rates in ICH patients (9). Lymphocytes serve as indicators of immune status, with studies indicating that lymphopenia at admission correlates with the increased infection risk and poor prognosis in spontaneous ICH patients (10). Additionally, platelet hyperactivity is associated with a higher risk of atherosclerotic vascular complications (11). In addition to single indicators, other composite indicators such as the Prognostic Nutrition Index (PNI), neutrophil-to-lymphocyte ratio (NLR), and platelet-to-lymphocyte ratio (PLR) have been shown to correlate with hematoma expansion and the prognosis of intracerebral hemorrhage (ICH) (12–14).

The HALP score serves as a comprehensive indicator that integrates nutritional, immune, and thrombotic-inflammatory pathways. Within this research framework, the HALP score has been introduced as a comprehensive biomarker. Previous studies have demonstrated its prognostic significance in various cancers (15, 16). Recent studies in the field of intensive care have demonstrated that the HALP score also reflects outcomes in sepsis (17), neurocritical care patients (18–20), and heart failure patients (21). In patients with acute ischemic stroke (AIS), a lower HALP score is associated with higher mortality risk (18, 19). Another study conducted among neurocritical care patients has suggested a negative correlation between the HALP score and short-term survival rates (20); however, these studies lacked external validation.

Current evidence regarding the relationship between the HALP score and the prognosis of ICH patients remains insufficient. To address this issue, this study aimed to investigate whether the HALP score can predict clinical outcomes, develop a predictive model based on this score, and validate the model’s performance in the external validation cohort.

## 2 Methods

### 2.1 Data source

This is a retrospective study using data from two distinct cohorts. The study was conducted in accordance with the Declaration of Helsinki. We accessed the MIMIC database under credit number 55670126, with all protected health information de-identified and informed consent waived. Data from the external validation cohort were collected from ICH patients admitted to the intensive care unit (ICU) at Hangzhou Lin’an Traditional Chinese Medicine Hospital from January 2022 to May 2025. Our study was approved by the hospital ethics committee (credit number LHTCM-II-Y-20251105-001). As this study was a retrospective study and did not involve the disclosure of patient privacy, the ethics committee exempted the requirement for informed consent.

### 2.2 Study population

The authors trained in clinical research evaluated and identified adult patients with a primary diagnosis of cerebral hemorrhage, including intracerebral and non-traumatic intracerebral hemorrhages in the hemispheres or intraventricular regions, from the MIMIC-IV database. These patients were considered eligible for study inclusion. Diagnoses used based on ICD-9 and ICD-10. The ICD codes are as follows: '431','1618','1611','1615','1619','1610','1614','169198','1612','169154','169151','1613','169120','1616','169192','169191','169122','169119','160193','169128','169131','169165','169118','169112','169159','16911','169111','169144','169134','169110','169121','169141','169153'.

The inclusion and exclusion criteria for the external validation cohort are identical to those of the internal cohort. The exclusion criteria included patients: (1) aged <18 years; (2) with repeat or no ICU admissions; (3) who died or stayed less than 24 h in the ICU; and (4) lacking key data such as hemoglobin, albumin, lymphocyte, and platelet counts. The final internal cohort comprised 925 patients, who were divided into four groups based on quartiles of the HALP score (Supplementary Figure 1).

### 2.3 Data collection

The data were extracted using SQL statements via PostgreSQL (version 17.8) and Navicat Premium (version 17.3.9). Data collection was performed within 24 h after ICU admission and included demographic data, laboratory tests, clinical severity scores, and interventions. Demographic data included age, sex, ethnicity, and weight. The recorded vital signs included temperature, respiratory rate, heart rate, mean arterial pressure, and oxygen saturation. Documented comorbidities comprised myocardial infarction, chronic obstructive pulmonary disease (COPD), congestive heart failure, severe liver disease, dementia, diabetes, malignant cancer, and sepsis. Laboratory data included measurements of white blood cell count (WBC), hemoglobin, platelet count, lymphocyte count, albumin, glucose, blood urea nitrogen, creatinine, potassium, sodium, chloride, and coagulation parameters. Treatment data encompassed mechanical ventilation, vasoactive agents, and renal replacement therapy (RRT).

Initial blood indicators were assessed upon ICU admission. Variables with missing values <20% were inputed using multiple imputation methods.

### 2.4 Clinical results

We obtained data on the length of stay (LOS) for both the hospital and ICU, along with the date of death (DOD). The MIMIC-IV database outcomes for intracerebral hemorrhage patients encompassed mortality rates during ICU stay, as well as at 30-, 90-, and 365-day mortality. The study’s main outcome was 30-day mortality. The secondary outcome endpoints were 90- and 365-day mortality.

### 2.5 Definitions of nutritional status

The HALP score was calculated using the formula: hemoglobin (g/L) × albumin (g/L) × lymphocytes (/L)/platelets (/L). Additionally, we considered two other nutritional status indicators: the Prognostic Nutritional Index (PNI) and the Geriatric Nutrition Risk Index (GNRI). The PNI was calculated as: PNI = albumin (g/L) + 5 × lymphocytes (10^9/L). The GNRI was calculated using the formula: GNRI = (14.89 × serum albumin [g/dL]) + (41.7 × [actual body mass index BMI/22 kg/m^2]), where 22 kg/m^2 is the designated ideal BMI (18).

### 2.6 Risk prediction modeling and statistical analysis

A normality test was performed on continuous variables. Data exhibiting a normal distribution were expressed as mean ± standard deviation (SD), whereas data with a non-normal distribution were presented as median (interquartile range, IQR) and frequency (percentage). Baseline characteristics were analyzed using the *t*-test or one-way analysis of variance (ANOVA) for continuous variables and Fisher’s exact test or Pearson’s chi-squared test for categorical variables. We used Kaplan–Meier curves to estimate cumulative incidences of all-cause mortality. We used both univariate and multivariate Cox regression analyses to evaluate the relationship between the HALP score and in-hospital mortality. RCS analyses were used to explore the dose–response relationship. Subgroup analyses were conducted for sex, age, and comorbidities.

Patients from the MIMIC database cohort were randomly assigned to training and testing cohorts in a 7:3 ratio. During the variable selection phase, the Boruta algorithm was first applied to identify relevant features. The variance inflation factor (VIF) was then calculated, and features with a VIF greater than 5 were excluded. Finally, multivariate Cox regression analysis was applied to the selected variables to estimate the final hazard ratios, and the prognostic model was constructed and visualized as a nomogram. Receiver operating characteristic (ROC) curves were used to evaluate model performance, and its performance was validated with an external cohort. Statistical analysis was performed using RStudio (version 4.4.3) and SPSS software (version 27.0), with a significance threshold set at a *p*-value of <0.05.

## 3 Results

### 3.1 Baseline characteristics

In the MIMIC-IV database, 925 intracerebral hemorrhage patients were analyzed, with 30-day, 90-day, and 365-day mortality rates of 29.08, 34.49, and 41.84%, respectively. The cohort had a mean age of 69 years (range: 57–81) with 55.35% male participants. Patients were categorized by HALP score quartiles at admission (Q1 < 16.87, Q2 16.87–34.27, Q3 34.27–44.89, Q4 > 44.89), and their baseline characteristics are detailed in Supplementary Table S1. The Q1 group exhibited a higher incidence of sepsis, increased use of vasoactive agents, and lower Glasgow Coma Scale (GCS) scores (*p* = 0.011, *p* < 0.001, and *p* = 0.045). The Q4 group exhibited a significantly higher proportion of male patients (*p* < 0.002) and greater body weight (*p* < 0.01). The Q4 group exhibited significantly lower Sequential Organ Failure Assessment (SOFA), APS III, and SAPS II scores than the Q1 group (*p* < 0.001 for all). The laboratory test results showed higher hemoglobin, lymphocyte counts, and albumin levels (*p* < 0.001 for all) as well as lower platelet counts and BUN levels in Q4 (*p* < 0.001 for both). Table 1 shows significant differences in mortality rates across different time frames. The Q1 group exhibited significantly higher values (30-day 39.83% vs. 90-day 35.50% vs. 365-day 34.05%, *p* < 0.001) than the other three groups.

**Table 1:** End-point events in patients across different groups.

Variables	Total (*n* = 925)	Q1 < 16.87 (*n* = 231)	Q2 16.87–34.27 (*n* = 231)	Q3 34.27–44.89 (*n* = 231)	Q4 > 44.89 (*n* = 232)	*p*-value
Events						
LOS ICU, day	4.66 (2.27, 9.78)	5.01 (2.43, 11.38)	5.05 (2.87, 9.81)	4.06 (1.98, 9.81)	4.02 (1.96, 8.33)	0.008
LOS hospital, day	8.98 (4.69, 16.74)	10.18 (5.08, 18.46)	9.99 (5.67, 16.41)	8.05 (4.60, 16.22)	7.64 (3.64, 15.88)	0.012
ICU-stay mortality, *n* (%)	223 (24.11)	75 (32.47)	57 (24.68)	41 (17.75)	50 (21.55)	0.002
30-day hospital mortality, *n* (%)	269 (29.08)	92 (39.83)	65 (28.14)	52 (22.51)	60 (25.86)	<0.001
90-day hospital mortality, *n* (%)	319 (34.49)	114 (49.35)	75 (32.47)	62 (26.84)	68 (29.31)	<0.001
365-day hospital mortality, *n* (%)	387 (41.84)	134 (58.01)	92 (39.83)	82 (35.50)	79 (34.05)	<0.001

### 3.2 Kaplan–Meier survival curve

**Figure 1:**
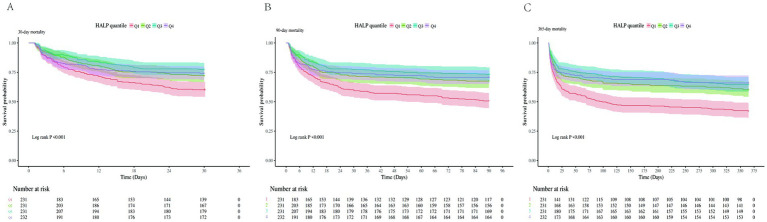
Kaplan–Meier curves illustrating all-cause mortality are presented for **(A)** 30-day, **(B)** 90-day, and **(C)** 365-day mortality.

It showed significant differences in survival probability among HALP quartile groups at 30, 90, and 365 days (*p* < 0.001), with the first quartile consistently showing the lowest survival probability (Figure 1).

### 3.3 Relationship between the HALP score and the risk of mortality

We developed three multivariate models to examine the relationship between various HALP classifications and all-cause mortality. Table 2 displays the hazard ratios (HRs) and 95% confidence intervals (CIs) for three models. Although consecutive HALP score was not significantly associated with the primary outcome, when analyzed as a categorical variable, compared with the first quartile group, the third quartile group showed significantly reduced mortality risks at 30, 90, and 365 days, with hazard ratios (HRs) of 0.72 (95% CI 0.50–1.00, *p* = 0.044), 0.67 (95% CI 0.48–0.93, *p* = 0.015), and 0.70 (95% CI 0.52–0.93, *p* = 0.014), respectively.

**Table 2:** Multivariable Cox regression analysis of the HALP score and all-cause mortality.

Variables	Model 1		Model 2		Model 3	
	HR (95%CI)	*p*	HR (95%CI)	*p*	HR (95%CI)	*p*
30-day mortality						
HALP	0.99 (0.99 ~ 1.00)	0.058	1.00 (0.99 ~ 1.00)	0.198	1.00 (1.00 ~ 1.01)	0.748
HALP quantile						
Q1	1.00 (reference)		1.00 (reference)		1.00 (reference)	
Q2	0.66 (0.48 ~ 0.90)	0.009	0.69 (0.50 ~ 0.95)	0.024	0.78 (0.56 ~ 1.10)	0.156
Q3	0.51 (0.36 ~ 0.72)	<0.001	0.56 (0.39 ~ 0.78)	<0.001	0.72 (0.50 ~ 1.00)	0.044
Q4	0.62 (0.44 ~ 0.85)	0.003	0.70 (0.50 ~ 0.98)	0.039	0.92 (0.65 ~ 1.29)	0.616
90-day mortality						
HALP	0.99 (0.99 ~ 0.99)	0.007	0.99 (0.99 ~ 0.99)	0.042	1.00 (0.99 ~ 1.00)	0.792
HALP quantile						
Q1	1.00 (reference)		1.00 (reference)		1.00 (reference)	
Q2	0.60 (0.45 ~ 0.80)	<0.001	0.62 (0.47 ~ 0.84)	0.002	0.74 (0.54 ~ 1.00)	0.045
Q3	0.48 (0.35 ~ 0.65)	<0.001	0.51 (0.37 ~ 0.70)	<0.001	0.67 (0.48 ~ 0.93)	0.015
Q4	0.55 (0.40 ~ 0.74)	<0.001	0.62 (0.45 ~ 0.84)	0.002	0.81 (0.59 ~ 1.12)	0.205
365-day mortality						
HALP	0.99 (0.99 ~ 0.99)	<0.001	0.99 (0.99 ~ 0.99)	0.005	0.99 (0.99 ~ 1.00)	0.289
HALP quantile						
Q1	1.00 (reference)		1.00 (reference)		1.00 (reference)	
Q2	0.60 (0.46 ~ 0.78)	<0.001	0.62 (0.47 ~ 0.81)	<0.001	0.73 (0.55 ~ 0.96)	0.024
Q3	0.51 (0.39 ~ 0.67)	<0.001	0.54 (0.41 ~ 0.72)	<0.001	0.70 (0.52 ~ 0.93)	0.014
Q4	0.51 (0.39 ~ 0.68)	<0.001	0.56 (0.42 ~ 0.75)	<0.001	0.74 (0.55 ~ 0.99)	0.047

Model 1: unadjusted.

Model 2: adjusted for age, sex, ethnicity, and weight.

Model 3: further adjusted for SOFA, SAPS II, GCS, CCI, ventilation status, vasoactive agents, liver disease, malignant cancer, temperature, MAP, and chloride.

**Figure 2:**
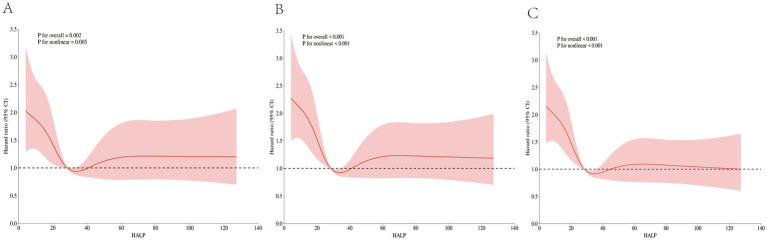
Relationship between the HALP score and the hazard ratio for all-cause mortality in ICH patients using a restricted cubic spline (RCS) curve. **(A)** 30-day, **(B)** 90-day, and **(C)** 365-day mortality.

An RCS curve was used to flexibly visualize and analyze the association between the HALP score and all-cause mortality in individuals with ICH. An L-shaped relationship exists between the HALP score and mortality, regardless of whether the patient is hospitalized or during follow-up (Figure 2). To further illustrate this association, Kaplan–Meier curves were generated (Supplementary Figure 2). A HALP score below 28.38 significantly elevates the 365-day mortality risk, with a declining HALP score associated with an increased risk (HR 0.651, 95% CI 0.532–0.797, *p* < 0.001).

### 3.4 Receiver operating characteristic analysis

**Figure 3:**
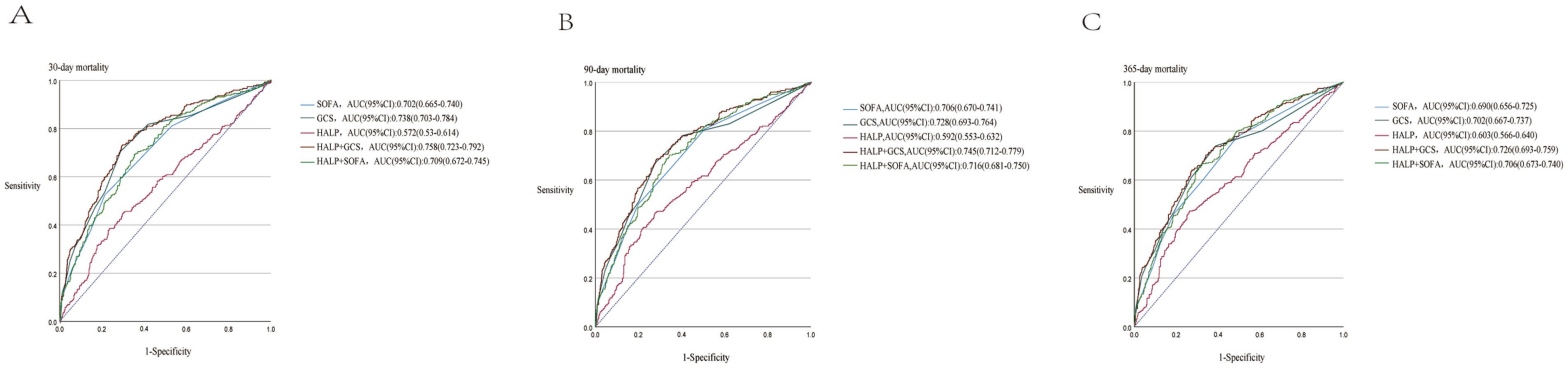
ROC curve for predicting all-cause mortality in ICH patients. **(A)** 30-day, **(B)** 90-day, and **(C)** 365-day mortality. The dashed line serves as the reference, indicating an ROC of 0.5.

ROC curves were generated for predicting all-cause death in patients at 30-day, 90-day, and 365-day follow-up after admission using six measures: SOFA, GCS, HALP, HALP+GCS, and HALP+SOFA scores (Figure 3).

Figure 3A illustrates that the AUC for the HALP score in predicting 30-day mortality in ICH patients was 0.572 (95% CI 0.53–0.614), while SOFA [0.702 (95% CI 0.665–0.740)] and GCS [0.738 (95% CI 0.703–0.784)] demonstrated superior prognostic accuracy. Combining the HALP score with common disease severity scores enhanced the predictive accuracy of the SOFA score (AUC 0.709 vs. 0.702) and the GCS score (AUC 0.758 vs. 0.738). This improvement was also evident during the 90-day and 365-day follow-up periods (Figures 3B,C). This indicates that HALP captures pathophysiological factors distinct from those assessed by existing tools and holds promise as a complementary biomarker.

### 3.5 Subgroup analyses

To further explore whether the relationships between the HALP score and short- and long-term all-cause mortality persisted in different conditions, subgroup analyses were conducted for sex, age, and comorbidities. A HALP score of 28.38 was used as the cutoff point to categorize patients into high HALP and low HALP groups for the subgroup analysis.

Supplementary Figure 3 shows the results of stratified analyses. Overall, although some subgroups did not show statistical significance, a HALP of <28.38 was associated with an increased risk of ICU mortality (Supplementary Figure 3A), 30-day (Supplementary Figure 3B), 90-day (Supplementary Figure 3C), and 365-day (Supplementary Figure 3D) all-cause mortality among different subgroups, except for CHF patients (interaction *p* for 365-day all-cause mortality = 0.048).

### 3.6 Feature selection and construction of the nomogram

Variables with *p*-values of >0.05 were excluded using a univariate logistic regression analysis because they are less likely to be relevant to clinical outcomes. The “Boruta” software package was used to screen for characteristic variables (Supplementary Figure 4), followed by the elimination of variables exhibiting strong multicollinearity (with VIF values ≥ 5).

**Figure 4:**
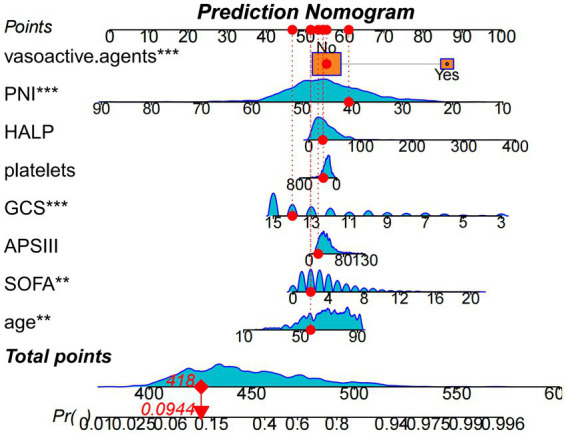
Nomogram to estimate the 30-day hospital mortality.

**Figure 5:**
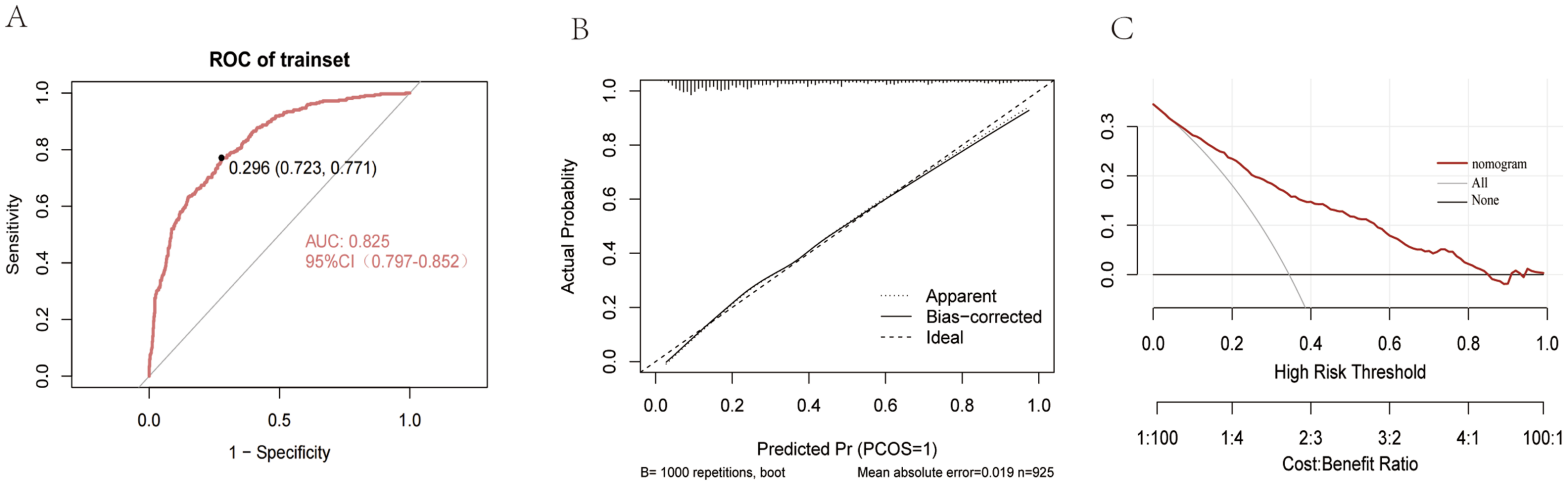
ROC curve, calibration, and decision curve analysis of the nomogram. **(A)** The nomogram demonstrates a discriminative performance with an AUROC value of 0.825. **(B)** The calibration curve. The solid line shows the bias-corrected calibration curve, and the dashed diagonal line represents perfect calibration. **(C)** Decision curve analysis was conducted for the nomogram. The horizontal axis represents the threshold probability, and the vertical axis represents the net benefit. The model is depicted by the brown curve, showing the net benefit of prediction-based decisions. The solid line “No intervention” indicates that no intervention was implemented, and thus no benefit was observed. The gray curve represents the scenario in which all samples received intervention.

Eight key predictive factors influencing 30-day all-cause mortality were ultimately identified: GCS, APS III, SOFA, PNI, HALP score, age, platelet count, and vasoactive agents, which were used to construct a risk prediction model for 30-day mortality in patients with intracerebral hemorrhage, as shown in Figure 4. The nomogram showed strong sensitivity and specificity in the internal cohort, achieving an AUROC of 0.825 (Figure 5A). The calibration curve (Figure 5B) was closely aligned with the ideal 45-degree line, demonstrating strong concordance between observed outcomes and predicted probabilities across the risk spectrum. The clinical usefulness of the nomogram was further validated using a decision curve analysis (Figure 5C). The model demonstrated a higher net benefit than default strategies across threshold probabilities ranging from approximately 50 to 80%.

### 3.7 External cohort verification

**Figure 6:**
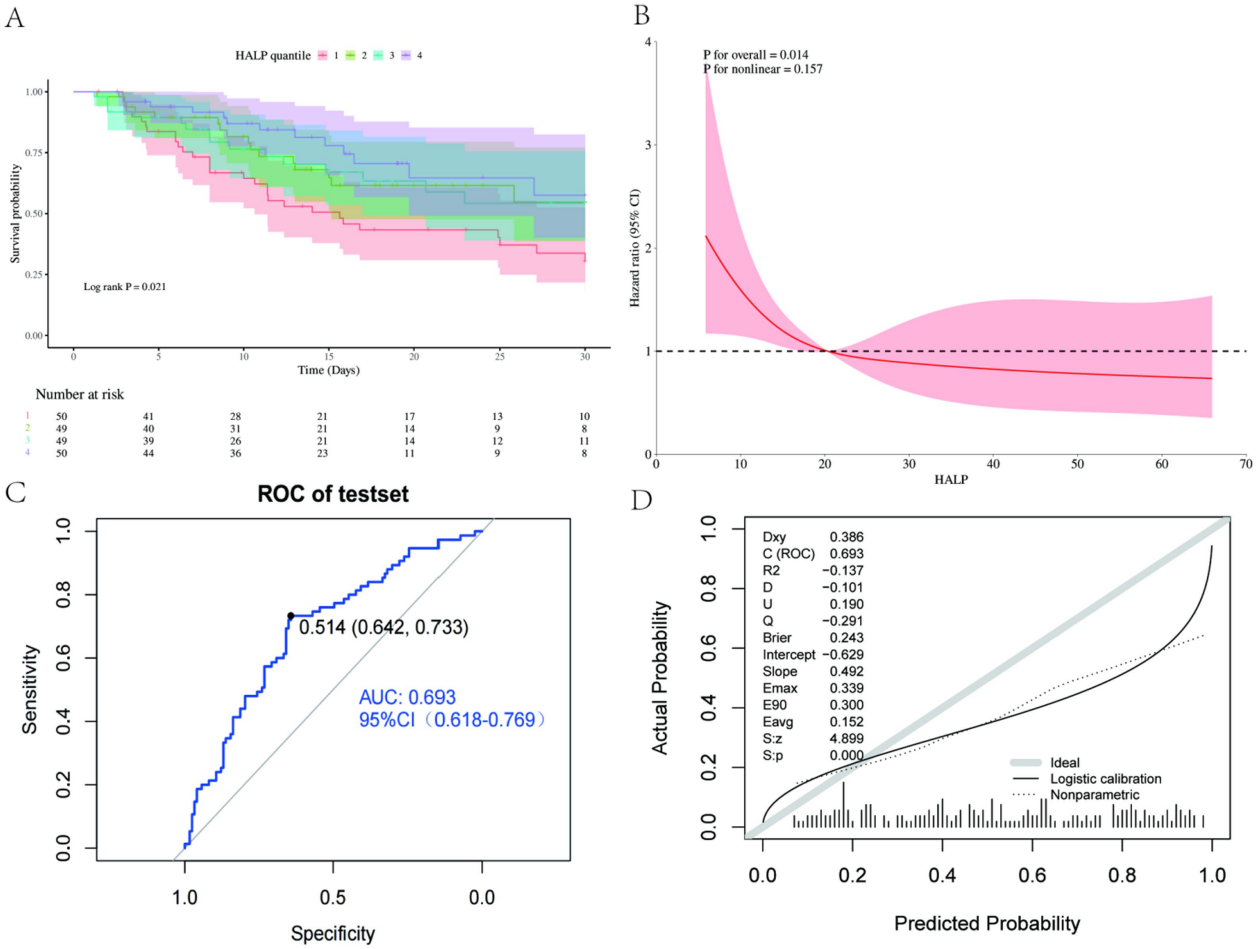
Correlation between the HALP score and mortality rates, along with the validation results of the nomogram in the external cohort. **(A)** K-M survival curve, **(B)** RCS curve, **(C)** ROC curve, and **(D)** calibration curve.

In an external cohort of 198 ICH patients with a 30-day mortality rate of 37.8%, individuals were categorized into quartiles (Q1–Q4) based on their HALP values at admission. The Kaplan–Meier analysis revealed that patients in Q1, who had lower HALP levels, exhibited significantly higher 30-day mortality (log-rank *p* = 0.021; Figure 6A). RCS analysis revealed a significant inverse dose–response relationship between the HALP score and mortality (*p* overall = 0.014; Figure 6B).

A comparison of characteristics between the two cohorts is presented in Supplementary Table 2. Although patients in the local dataset exhibited more severe conditions (as evidenced by lower GCS scores and higher 30-day mortality rates), this prognostic nomogram still showed excellent performance, with an AUC value of 0.693 (95% CI: 0.618–0.769) (Figure 6C) and good calibration between predicted and observed mortality probabilities (Figure 6D).

## 4 Discussion

The analyses indicated that a lower HALP score at admission was significantly associated with a higher risk of adverse outcomes. The restricted cubic spline curve analysis revealed an L-shaped relationship between HALP levels and primary adverse outcomes. These results, confirmed by our external cohort, indicate that the HALP score could be a novel, easily accessible exploratory biomarker associated with clinical outcomes in ICH patients. Furthermore, we developed a nomogram as a straightforward and effective risk stratification tool, aiding clinicians in identifying at-risk individuals for timely intervention.

Intracerebral hemorrhage is a prevalent stroke syndrome that significantly impacts global healthcare systems, necessitating prompt evaluation. Recent advancements highlight the potential of blood biomarkers detected upon admission for effective diagnosis and prognostic assessment of this condition (22, 23). Our study suggests that the HALP score may serve as a novel composite biomarker independently associated with both short-term and long-term mortality in patients with intracerebral hemorrhage. We further found that patients with a low HALP score had a higher incidence of sepsis, increased use of vasoactive agents, and lower GCS, SOFA, and APS III scores. Kaplan–Meier curves indicated significant differences in survival probabilities across HALP quartiles at 30, 90, and 365 days (*p* < 0.001), with the first quartile (HALP < 16.87) consistently showing the lowest survival probability.

Following intracerebral hemorrhage, neuroinflammation can cause substantial harm to brain tissue and potentially result in immunosuppression, correlating with unfavorable clinical results (4, 5, 7). Inflammatory activation typically induces coagulation disorders. Research has indicated that lymphocytes, neutrophils, and white blood cells are key indicators of acute coagulation dysfunction in ICH patients (13, 14). This inflammatory response triggers thrombosis, involving platelet adhesion, release, and aggregation (24). The disruption of the platelet aggregation balance, which is crucial for hemostasis, can lead to coagulation disorders and platelet dysfunction, potentially worsening ICH (25). Additionally, a meta-analysis has suggested that anemia and thrombosis may intensify inflammation. Anemia at admission is associated with increased mortality and adverse prognosis risk in patients with ICH (26).

In addition, malnutrition is closely associated with weakened immune function, increased risk of infection, and elevated mortality rates in severe cases (6, 8). Albumin provides neuroprotection by mitigating oxidation, stasis, thrombosis, and leukocyte adhesion (27). Hypoalbuminemia, often seen as a marker of malnutrition, also indicates a systemic inflammatory response. Several commonly used clinical indicators, such as the Controlled Nutritional Status (CONUT) score, the Prognostic Nutrition Index (PNI), and the Geriatric Nutrition Index (GNRI), incorporate serum albumin levels. Numerous studies (8, 12, 28, 29) have confirmed the prognostic significance of these assessment methods and their association with clinical outcomes in patients with cardiovascular and cerebrovascular diseases.

The prognostic value of HALP likely stems from its holistic evaluation of systemic inflammation and nutritional status. Its prognostic value in patients with acute ischemic stroke has been extensively reported. A prospective observational study (19) conducted in 2024 found that a lower HALP score was associated with increased mortality in stroke patients. Tian et al. (18) found that a higher HALP score at admission was associated with reduced risk of stroke recurrence and lower mortality rates within 90 days and 1 year. The addition of the HALP score to conventional risk factors enhances the ability to distinguish recurrent stroke from death and facilitates risk reclassification, suggesting that a decreased HALP score may be an independent risk factor for stroke recurrence and mortality. A recently published study evaluated the prognostic value of HALP in patients with acute ischemic stroke (30). Patients with lower HALP scores at admission are at higher risk for prolonged hospital stay, need for intensive care, and mortality, suggesting that a low HALP score may indicate neuroinflammation and metabolic exhaustion, impairing recovery from neurological injury.

Patients suffering from cerebral hemorrhage exhibit cerebral hypoperfusion and neurological deficits. The etiology of hemorrhagic stroke and ischemic stroke involves oxidative stress, hemostasis, and inflammation. While numerous studies have investigated the association between the HALP score and ischemic stroke outcomes, research on the prognostic significance of the HALP score in patients with cerebral hemorrhage remains scarce. In our study, the HALP score not only independently predicted outcomes but also enhanced prognostic accuracy when combined with traditional severity scores, improving the predictive accuracy of the SOFA score (AUC 0.709 vs. 0.702) and the GCS score (AUC 0.758 vs. 0.738). This indicates that it captures pathophysiological factors distinct from those assessed by existing tools and holds promise as a complementary biomarker. It is worth noting that these findings are consistent with Zou’s study (20), which included all critically ill patients with brain injury, both hemorrhagic and ischemic. In contrast, our research specifically limits the study population to intracerebral hemorrhage, thereby better highlighting the characteristics within this subgroup.

Furthermore, we have devised a prognostic model using the HALP score to predict the risk of 30-day mortality in patients with ICH. This model is depicted in a user-friendly nomogram design, facilitating the evaluation of mortality risk through the use of commonly collected clinical information. The model was validated with an independent cohort, achieving an AUC of 0.825 (95%CI: 0.797–0.852) in the training cohort and 0.693 (95%CI: 0.618–0.769) in the validation cohort. Healthcare providers can quickly assess mortality risk in ICU-admitted ICH patients, providing actionable insights for personalized clinical decision-making.

We developed a simplified nomogram model by integrating eight risk factors, all of which have been previously associated with mortality risk in ICH patients. Disease severity scores are effective predictors of 1-year mortality, as supported by Aytuluk’s research (31), which found them superior to functional outcome scores despite lacking critical radiological parameters for ICH. Zhu et al. (32) have demonstrated that a GCS score of 13 to 15 at admission correlates with a favorable 7-day outcome. Our study also identified advanced age and low platelet count as significant risk factors for poor outcomes, consistent with existing research (33). Furthermore, the Prognostic Nutritional Index, which indicates both nutritional and immune status, has been validated as a standalone prognostic determinant for complications and overall survival among individuals with spontaneous intracerebral hemorrhage through several clinical investigations (12).

This study’s primary strength lies in its pioneering use of a large dataset to examine the impact of HALP on both short- and long-term mortality in ICU patients with ICH. It includes survival analysis, Cox regression, nomogram construction, and external validation. HALP’s simplicity and accessibility enhance its value in clinical practice for identifying patients at higher mortality risk. However, further research should address the limitations in this study. First, the retrospective cohort study’s single-center design inherently introduces selection bias. To address this, we adjusted the covariates to improve the accuracy of the results and validated them externally with a real-world cohort. However, these findings are exploratory, and the generalizability is limited, necessitating further validation in prospective, multi-center cohorts. Second, this study examined only the baseline HALP value at ICU admission, without considering its dynamic changes during hospitalization; future research should investigate the predictive value of a series of HALP measurements or trajectory changes. Third, patients who died within the first 24 h of ICU admission were excluded from the analysis. This exclusion could potentially affect the generalizability of the findings, as the sickest patients may not have been represented in the final cohort; therefore, our findings cannot be generalized to this specific subgroup of critically ill patients. Last but not least, our study identified ICH patients using the ICD-9/10 coding system. Although standardized coding is a widely adopted method in large-scale database research, classification bias may still exist. The database lacks detailed neurological imaging data, such as hematoma volume, location, and extent of intracranial extension. As these factors are known to be important determinants of prognosis in patients with intracerebral hemorrhage, their unavailability may have influenced the observed association between the HALP score and mortality. Further validation using prospectively confirmed cases of intracerebral hemorrhage and inclusion of imaging parameters are still required.

## 5 Conclusion

This study confirmed that a low HALP score is associated with increased mortality in patients with intracerebral hemorrhage. Furthermore, the established predictive model demonstrated that integrating this composite biomarker of immunonutritional status could serve as an effective tool for predicting poor prognosis in ICH patients.

## Data Availability

The data analyzed in this study is subject to the following licenses/restrictions: The datasets used and/or analyzed during the current study are available from the corresponding author on reasonable request. Requests to access these datasets should be directed to J-TG, sarahgjt@163.com.
