# Supplementary material for: The impact of early nutritional and immune status assessment on all-cause mortality in patients with intracerebral hemorrhage in the intensive care unit: a retrospective study
Source: Front Med (Lausanne). 2026 Jul 7;13:1852107. doi: 10.3389/fmed.2026.1852107 (PMC13385231; doi:10.3389/fmed.2026.1852107)
Supplement: Supplementary file 1 [file Supplementary_file_1.docx]

Supplementary Material

# Supplementary Figures and Tables

## Supplementary Figures


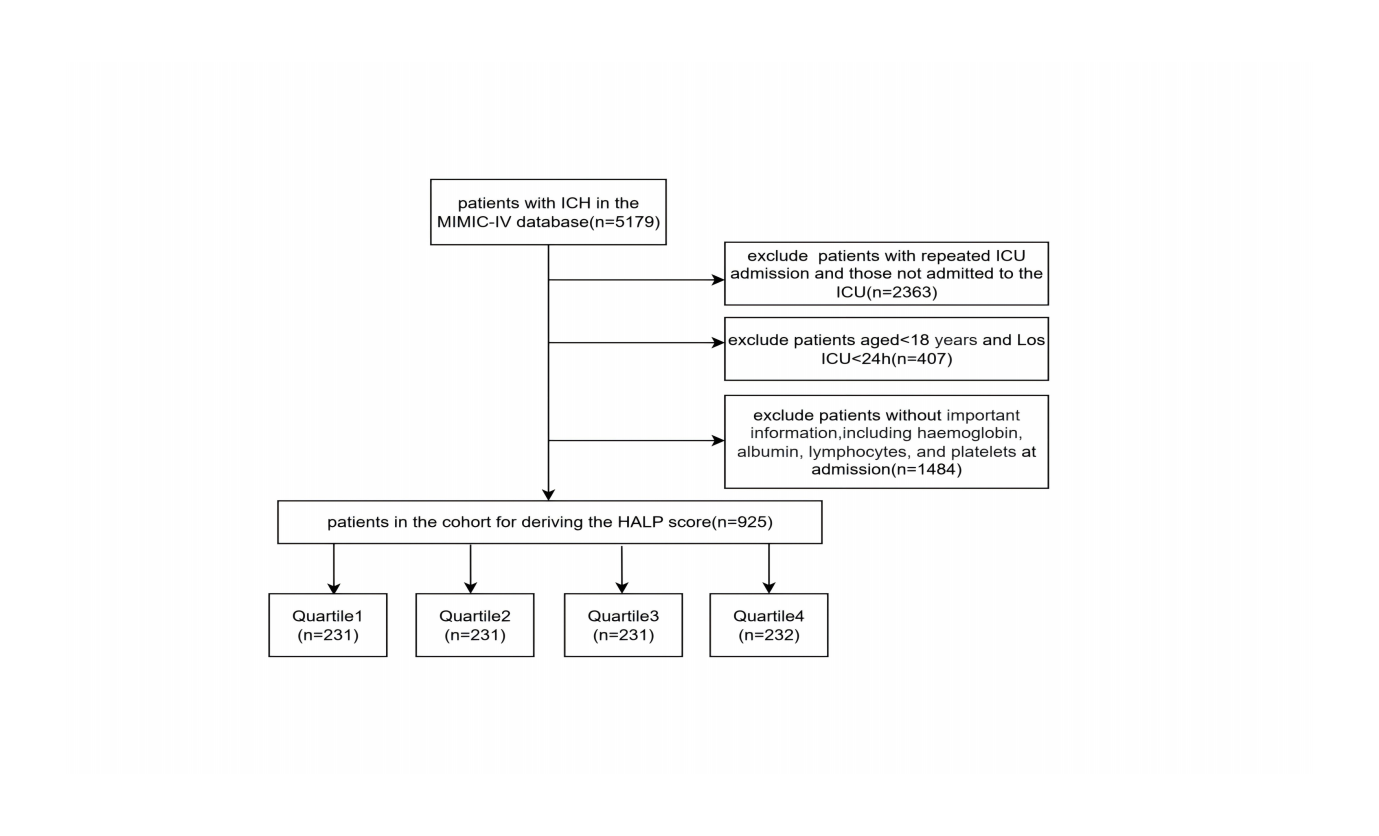


Figure1. Flowchart of patients selection

**
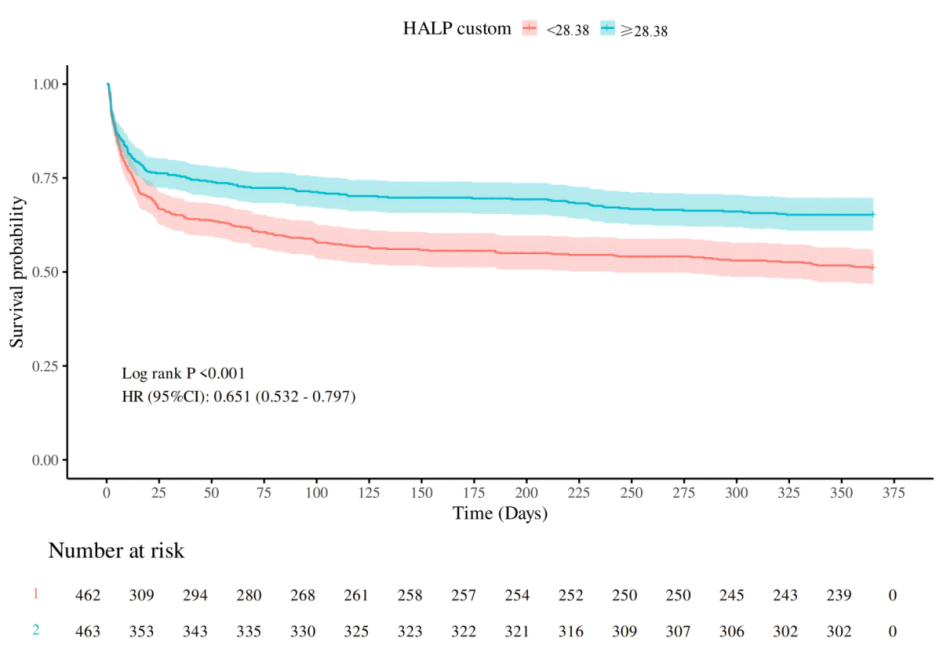
**

**Figure2.**The Kaplan–Meier survival curves for 365-day mortality stratified by low and high HALP level

**
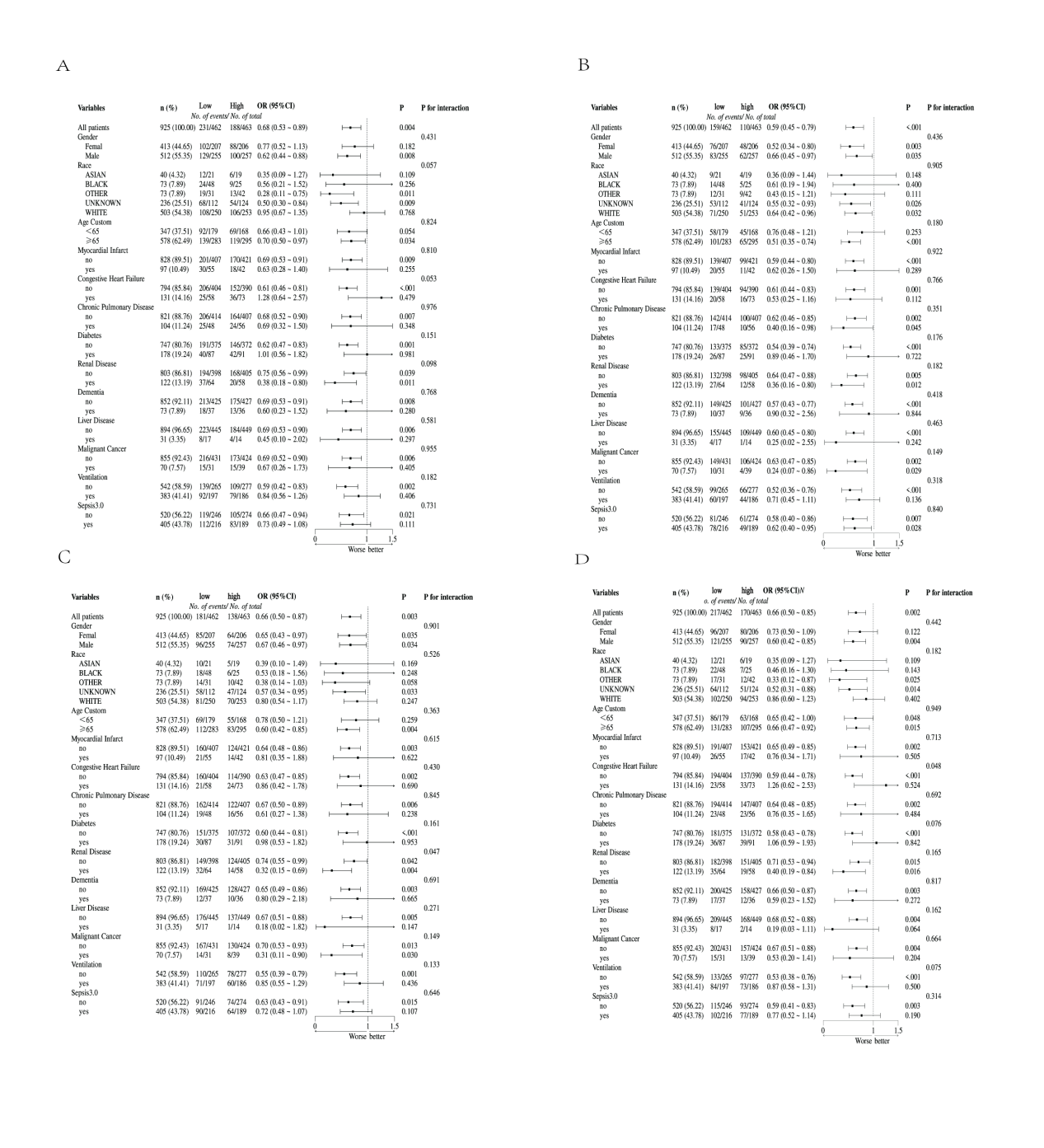
**

**Figure3 Forest plots for all-cause mortality by subgroup, accounting for gender, age, and comorbidities: (A) ICU-day, (B) 30-day, (C) 90-day,and (D)365-day. CI,confidence interval.**


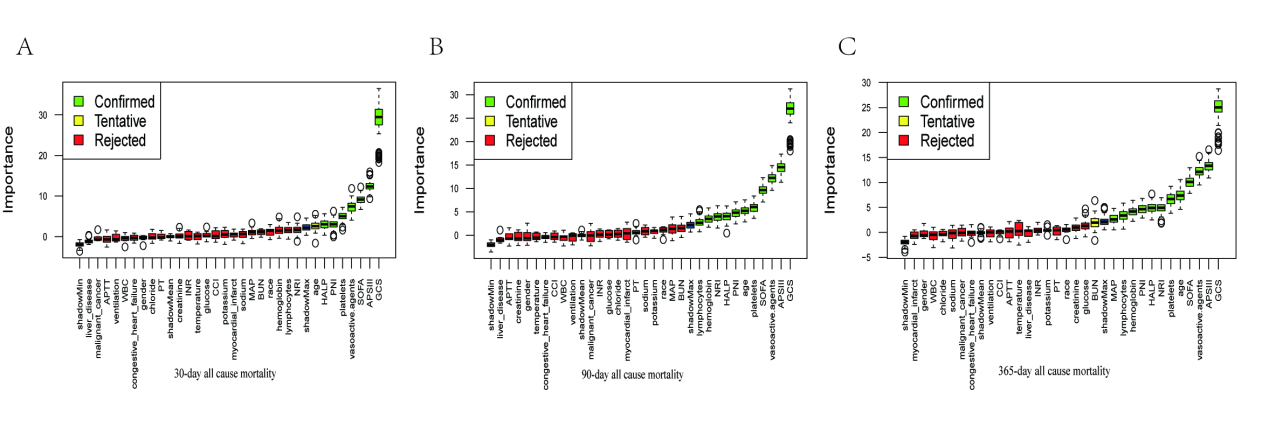


**Figure4**.Boruta feature selection was conducted to evaluate clinically relevant covariates in relation to the primary outcomes: (a) 30-day, (b) 90-day, and (c) 365-day.The Boruta algorithm was employed to rank the importance of the selected variables.A bar chart displays feature significance using color coding: green for important features, red for unimportant ones, and yellow for tentative features.

## Supplementary Tables

**Supplementary Table 1.**:Baseline characteristics stratified by HALP quartiles.

| Variables | Total  (n = 925) | Q1<16.87  (n = 231) | Q2  16.87-34.27 (n = 231) | Q3  34.27-44.89  (n = 231) | Q4>44.89  (n = 232) | *p-Value* |
| --- | --- | --- | --- | --- | --- | --- |
|  |  |  |  |  |  |  |
| **Demographic** |  |  |  |  |  |  |
| Age, years | 69.00 (57.00, 81.00) | 71.00 (60.00,81.00) | 71.00 (60.00,81.00) | 68.00 (57.00,80.50) | 68.50 (56.75,78.00) | 0.222 |
| Gender, n(%) |  |  |  |  |  | **0.002** |
| Female | 413 (44.65) | 125 (54.11) | 104 (45.02) | 99 (42.86) | 85 (36.64) |  |
| Male | 512 (55.35) | 106 (45.89) | 127 (54.98) | 132 (57.14) | 147 (63.36) |  |
| Race, n(%) |  |  |  |  |  | 0.130 |
| ASIAN | 40 (4.32) | 11 (4.76) | 7 (3.03) | 6 (2.60) | 16 (6.90) |  |
| BLACK | 73 (7.89) | 12 (5.19) | 18 (7.79) | 22 (9.52) | 21 (9.05) |  |
| WHITE | 503 (54.38) | 120 (51.95) | 129 (55.84) | 130 (56.28) | 124 (53.45) |  |
| UNKNOWN | 236 (25.51) | 71 (30.74) | 63 (27.27) | 54 (23.38) | 48 (20.69) |  |
| OTHER | 73 (7.89) | 17 (7.36) | 14 (6.06) | 19 (8.23) | 23 (9.91) |  |
| Weight,Kg | 76.20 (63.50, 90.00) | 70.55 (58.20,86.17) | 75.40 (64.00,90.05) | 77.00 (64.60,89.30) | 80.00 (70.00,92.43) | **<.001** |
| **Vital signs** |  |  |  |  |  |  |
| Temperature,℃ | 36.97 (36.75, 37.28) | 36.98 (36.77,37.26) | 36.95 (36.76,37.27) | 36.95 (36.72,37.28) | 36.99 (36.75,37.31) | 0.643 |
| Heart Rate,bmp | 80.92 (71.53, 89.89) | 82.18 (71.78,90.18) | 81.80 (73.59,91.19) | 79.00 (69.72,89.79) | 79.73 (71.85,88.88) | 0.361 |
| Resp Rate, bmp | 18.66 (16.85, 20.86) | 18.45 (16.93,20.44) | 18.73 (17.11,20.96) | 18.54 (16.49,20.96) | 18.75 (16.69,20.84) | 0.642 |
| SBP, mmHg, | 129.35 ± 14.12 | 129.80 ± 14.92 | 127.37 ± 12.98 | 129.99 ± 14.71 | 130.23 ± 13.68 | 0.104 |
| DBP, mmHg, | 68.83 ± 11.59 | 67.82 ± 11.68 | 68.45 ± 11.23 | 69.37 ± 12.19 | 69.69 ± 11.22 | 0.287 |
| SpO2, | 97.24 (96.00, 98.71) | 97.36 (96.20,98.87) | 97.00 (95.82,98.66) | 97.48 (96.05,98.94) | 97.15 (96.00,98.56) | 0.445 |
| Urineoutput,ml, | 1537.50 (923.75, 2200.00) | 1500.00 (876.00,2142.50) | 1492.50 (888.00,2222.50) | 1558.00 (1097.50,2125.00) | 1580.00 (918.75,2313.75) | 0.580 |
| **Comorbidities** |  |  |  |  |  |  |
| Myocardial Infarct, n(%) | 97 (10.49) | 25 (10.82) | 28 (12.12) | 21 (9.09) | 23 (9.91) | 0.742 |
| Congestive Heart Failure, n(%) | 131 (14.16) | 30 (12.99) | 35 (15.15) | 31 (13.42) | 35 (15.09) | 0.869 |
| COPD, n(%) | 104 (11.24) | 27 (11.69) | 25 (10.82) | 24 (10.39) | 28 (12.07) | 0.937 |
| Diabetes, n(%) | 178 (19.24) | 31 (13.42) | 48 (20.78) | 51 (22.08) | 48 (20.69) | 0.075 |
| Renal Disease, n(%) | 122 (13.19) | 31 (13.42) | 27 (11.69) | 33 (14.29) | 31 (13.36) | 0.870 |
| Liver Disease, n(%) | 31 (3.35) | 10 (4.33) | 8 (3.46) | 8 (3.46) | 5 (2.16) | 0.632 |
| Dementia, n(%) | 73 (7.89) | 16 (6.93) | 16 (6.93) | 19 (8.23) | 22 (9.48) | 0.697 |
| Malignant Cancer, n(%) | 70 (7.57) | 21 (9.09) | 15 (6.49) | 15 (6.49) | 19 (8.19) | 0.647 |
| Sepsis3, n(%) | 405 (43.78) | 118 (51.08) | 83 (35.93) | 99 (42.86) | 105 (45.26) | **0.011** |
| **Severity scores** |  |  |  |  |  |  |
| SOFA, | 3.00 (2.00, 5.00) | 4.00 (2.00,6.00) | 3.00 (2.00,5.00) | 3.00 (1.00,5.00) | 3.00 (2.00,5.00) | **<.001** |
| APSIII, | 37.00 (28.00, 48.00) | 42.00 (33.00,56.00) | 37.00 (28.00,46.00) | 34.00 (26.50,46.00) | 34.00 (26.75,47.25) | **<.001** |
| SAPSII, | 33.00 (26.00, 41.00) | 36.00 (30.00,45.00) | 33.00 (27.00,41.50) | 31.00 (24.00,39.00) | 31.00 (24.75,39.00) | **<.001** |
| GCS, | 13.00 (10.00, 15.00) | 12.00 (9.00,14.00) | 13.00 (10.00,15.00) | 13.00 (11.00,15.00) | 13.00 (10.75,15.00) | **0.045** |
| Charlson Comorbidity Index, | 6.00 (4.00, 8.00) | 6.00 (4.00,7.00) | 6.00 (4.00,8.00) | 6.00 (4.00,8.00) | 6.00 (4.00,8.00) | 0.667 |
| **Laboratory parameters** |  |  |  |  |  |  |
| WBC,K/µL, | 10.30 (8.25, 13.50) | 10.60 (8.45,13.95) | 10.05 (7.95,13.30) | 10.25 (8.22,12.90) | 10.35 (8.35,13.70) | 0.480 |
| Lymphocytes,K/µL, | 1.18 (0.79, 1.70) | 0.66 (0.47,0.91) | 0.95 (0.79,1.19) | 1.39 (1.09,1.71) | 1.85 (1.52,2.43) | **<.001** |
| Hemoglobin,g/dL, | 12.42 ± 2.02 | 11.21 ± 2.03 | 12.21 ± 1.78 | 12.84 ± 1.84 | 13.41 ± 1.76 | **<.001** |
| Platelets,K/µL, | 201.50 (157.00, 258.00) | 254.50 (176.00,319.00) | 200.00 (166.50,251.00) | 202.50 (158.00,243.25) | 173.75 (142.00,214.62) | **<.001** |
| Albumin,g/dL, | 3.90 (3.50, 4.30) | 3.60 (3.10,3.90) | 3.90 (3.40,4.20) | 4.10 (3.70,4.35) | 4.10 (3.90,4.40) | **<.001** |
| Glucose,mg/dL, | 129.00 (108.75, 153.80) | 130.00 (108.78,155.38) | 130.00 (109.90,155.67) | 127.50 (109.20,150.75) | 128.75 (107.47,151.00) | 0.869 |
| BUN,mg/dL, | 16.50 (12.50, 23.00) | 17.50 (13.25,25.00) | 17.00 (12.75,23.00) | 15.00 (12.00,20.00) | 16.00 (12.00,21.50) | **0.010** |
| Creatinine,mg/dL, | 0.90 (0.75, 1.15) | 0.95 (0.75,1.23) | 0.90 (0.70,1.17) | 0.90 (0.70,1.10) | 0.90 (0.75,1.15) | 0.174 |
| Potassium,mmol/L | 4.00 (3.70, 4.35) | 4.00 (3.75,4.38) | 4.00 (3.67,4.35) | 4.00 (3.75,4.20) | 3.95 (3.70,4.31) | 0.356 |
| Sodium, mmol/L | 139.83 ± 4.64 | 139.71 ± 5.11 | 140.37 ± 4.73 | 139.57 ± 4.32 | 139.68 ± 4.34 | 0.238 |
| Chloride, mmol/L | 103.92 ± 5.21 | 103.59 ± 5.26 | 104.49 ± 5.30 | 103.77 ± 4.83 | 103.81 ± 5.41 | 0.269 |
| INR, | 1.15 (1.05, 1.30) | 1.15 (1.05,1.30) | 1.15 (1.05,1.25) | 1.15 (1.05,1.30) | 1.15 (1.05,1.30) | 0.916 |
| PT,s, | 12.50 (11.60, 14.15) | 12.55 (11.65,14.22) | 12.50 (11.53,13.90) | 12.55 (11.65,14.30) | 12.50 (11.60,14.10) | 0.895 |
| PTT,s, | 28.50 (25.65, 31.60) | 28.50 (25.58,31.55) | 28.60 (25.50,31.70) | 28.45 (25.70,31.45) | 28.55 (26.09,31.81) | 0.780 |
| PNI, | 45.62 (40.35, 50.00) | 38.65(34.54,43.20) | 44.00(39.80,47.30) | 47.50(44.68,50.58) | 51.38(47.74,54.50) | **<.001** |
| GNRI, | 110.60 (100.56, 121.08) | 102.83 (93.74,112.84) | 109.03 (100.42,120.66) | 113.49 (102.95,123.96) | 115.39 (108.20,123.46) | **<.001** |
| **Treatments** |  |  |  |  |  |  |
| Vasoactive Agents, n(%) | 157 (16.97) | 61 (26.41) | 32 (13.85) | 35 (15.15) | 29 (12.50) | **<.001** |
| Ventilation, n(%) | 383 (41.41) | 102 (44.16) | 97 (41.99) | 88 (38.10) | 96 (41.38) | 0.616 |
| CRRT, n(%) | 26 (2.81) | 4 (1.73) | 9 (3.90) | 5 (2.16) | 8 (3.45) | 0.444 |

SBP, Systolic blood pressure; DBP,Diastolic Blood Pressure;SpO2, Oxygen saturation; GCS,Glasgow Coma Scale;SOFA,Sequential organ failure assessment; APS III, Acute Physiology Score III;SAPSII,Simplified Acute Physiology Score II;COPD,Chronic Pulmonary Disease;WBC, White blood cell count; BUN, blood urea nitrogen;INR, International normalized ratio; PT,Prothrombin Time;PTT,activated partial thromboplastin time;CRRT, Continuous renal replacement therapy;PNI,Prognostic Nutritional Index;GNRI,Geriatric nutrition Risk Index.

The variables with bold p-values are statistically significant.

**Supplementary Table2 Comparison of characteristics between two different cohorts**

| Variables | Total (n = 1123) | Mimic cohort (n = 925) | external cohort (n = 198) | Statistic | *P* |
| --- | --- | --- | --- | --- | --- |
|  |  |  |  |  |  |
| Age, M (Q₁, Q₃) | 69.00 (57.00, 80.00) | 69.00 (57.00, 81.00) | 69.00 (55.25, 79.00) | Z=-0.52 | 0.603 |
| SOFA, M (Q₁, Q₃) | 3.00 (2.00, 5.00) | 3.00 (2.00, 5.00) | 3.00 (2.00, 4.00) | Z=-0.85 | 0.394 |
| APSIII, M (Q₁, Q₃) | 37.00 (28.00, 48.00) | 37.00 (28.00, 48.00) | 36.00 (28.00, 49.00) | Z=-0.12 | 0.901 |
| GCS, M (Q₁, Q₃) | 13.00 (10.00, 15.00) | 13.00 (10.00, 15.00) | 11.00 (6.25, 14.00) | Z=-6.78 | <.001 |
| Platelets, M (Q₁, Q₃) | 199.00 (153.50, 256.00) | 201.50 (157.00, 258.00) | 184.50 (138.25, 238.75) | Z=-2.91 | 0.004 |
| HALP, M (Q₁, Q₃) | 27.20 (16.01, 43.23) | 28.38 (16.87, 44.89) | 20.30 (13.59, 34.07) | Z=-4.52 | <.001 |
| PNI, M (Q₁, Q₃) | 44.70 (38.97, 49.50) | 45.62 (40.35, 50.00) | 39.30 (34.61, 44.06) | Z=-9.57 | <.001 |
| Vasoactive Agents, n(%) | 213 (18.97) | 157 (16.97) | 56 (28.28) | χ²=13.57 | <.001 |
| 30-day mortality, n(%) | 344 (30.63) | 269 (29.08) | 75 (37.88) | χ²=5.94 | 0.015 |
| Los Icu, M (Q₁, Q₃) | 4.80 (2.42, 9.95) | 4.66 (2.27, 9.78) | 5.82 (3.00, 10.87) | Z=-2.32 | 0.020 |
| Los Hospital, M (Q₁, Q₃) | 9.78 (4.87, 18.03) | 8.98 (4.69, 16.74) | 12.94 (7.03, 22.75) | Z=-4.48 | <.001 |

GCS,Glasgow Coma Scale;SOFA,Sequential organ failure assessment; APS III, Acute Physiology Score III;PNI,Prognostic Nutritional Index

**R code**

**#####Collinearity Check**

**vif_data <- my_dt %>% mutate(status = ifelse(status=='1' ,1 ,0 ))**

**independent_vars <- c("APSIII","SAPSII","SOFA","GCS","CCI")**

**formula <- status~ APSIII+SAPSII+SOFA+GCS+CCI**

**vif_model <- glm(formula, data = my_dt, family=binomial)**

**summary(vif_model)**

**vif_values <- car::vif(vif_model)**

**print(vif_values)**

**#####Boruta**

**mydata=as.data.frame(lapply(mydata,function(x)**

**{if(is.factor(x)){as.numeric(x)}else{x}}))**

**boruta_output=Boruta(status~.,**

**data=mydata,**

**doTrace=2)**

**par(mar=c(8,5,3,3)+0.1)**

**plot(boruta_output,las=2,**

**cex.axis=0.7,xlab=NULL,ylab="Importance",**

**main="Boruta Feature Importance",**

**pin=c(10,8))**

**ddist <- datadist(data)**

**options(datadist = "ddist")**

**##### Create a logistic regression model**

**fit <- lrm(status90 ~ age + SOFA + APSIII +**

**GCS + platelets + HALP +**

**PNI + vasoactive.agents,**

**data = data)**

**print(summary(fit))**

**nomogram <- regplot(**

**fit,**

**plots = c('violin','boxes'), observation = data[4,], center = TRUE, subticks = TRUE, droplines = TRUE, title = 'Dynamic Nomogram', points = TRUE, odds = TRUE, showP = TRUE, rank = 'sd', interval = 'confidence', clickable = FALSE )**
